# Compositional Differences in the Oral Microbiome of E-cigarette Users

**DOI:** 10.3389/fmicb.2021.599664

**Published:** 2021-05-31

**Authors:** Jessica Chopyk, Christine M. Bojanowski, John Shin, Alex Moshensky, Ana Lucia Fuentes, Saniya S. Bonde, Dagni Chuki, David T. Pride, Laura E. Crotty Alexander

**Affiliations:** ^1^Department of Pathology, University of California San Diego (UCSD), La Jolla, CA, United States; ^2^Section of Pulmonary Diseases, Critical Care and Environmental Medicine, Department of Medicine, Tulane University, New Orleans, LA, United States; ^3^Pulmonary Critical Care Section, VA San Diego Healthcare System, La Jolla, CA, United States; ^4^Division of Pulmonary, Critical Care and Sleep Medicine, Department of Medicine, University of California San Diego (UCSD), La Jolla, CA, United States; ^5^Department of Medicine, University of California San Diego (UCSD), La Jolla, CA, United States

**Keywords:** e-cigarette, vaping, oral microbiome, *Staphylococcus*, 16S

## Abstract

Electronic (e)-cigarettes have been advocated as a safer alternative to conventional tobacco cigarettes. However, there is a paucity of data regarding the impact of e-cigarette aerosol deposition on the human oral microbiome, a key component in human health and disease. We aimed to fill this knowledge gap through a comparative analysis of the microbial community profiles from e-cigarette users and healthy controls [non-smokers/non-vapers (NSNV)]. Moreover, we sought to determine whether e-cigarette aerosol exposure from vaping induces persistent changes in the oral microbiome. To accomplish this, salivary and buccal mucosa samples were collected from e-cigarette users and NSNV controls, with additional oral samples collected from e-cigarette users after 2 weeks of decreased use. Total DNA was extracted from all samples and subjected to PCR amplification and sequencing of the V3-V4 hypervariable regions of the 16S rRNA gene. Our analysis revealed several prominent differences associated with vaping, specific to the sample type (i.e., saliva and buccal). In the saliva, e-cigarette users had a significantly higher alpha diversity, observed operational taxonomic units (OTUs) and Faith’s phylogenetic diversity (PD) compared to NSNV controls, which declined with decreased vaping. The buccal mucosa swab samples were marked by a significant shift in beta diversity between e-cigarette users and NSNV controls. There were also significant differences in the relative abundance of several bacterial taxa, with a significant increase in *Veillonella* and *Haemophilus* in e-cigarette users. In addition, nasal swabs demonstrated a trend toward higher colonization rates with *Staphylococcus aureus* in e-cigarette users relative to controls (19 vs. 7.1%; *p* = n.s.). Overall, these data reveal several notable differences in the oral bacterial community composition and diversity in e-cigarette users as compared to NSNV controls.

## Introduction

Electronic (e)-cigarettes and other vaping devices work by battery operated coils heating and aerosolizing e-liquids into an inhalable cloud of chemicals. E-liquids most often contain propylene glycol, glycerin, nicotine, and flavoring chemicals, but can also contain THC, metals, and other substances. They first became widely available in the United States in 2007 and were marketed as being safer than traditional cigarettes (Farsalinos et al., 2015; National Center for Health Statistics, 2017). Over 2014–2018, daily e-cigarette use increased dramatically among adolescents and young adults, corresponding with increased marketing and the introduction of appealing flavors (Orellana-Barrios et al., 2015; Romberg et al., 2019). According to the United States Surgeon General, approximately 1.5 million more youths used e-cigarettes in 2018 (3.6 million), compared with 2017 (2.1 million; Gentzke et al., 2019). Given the lack of data on long-term effects and safety of e-cigarettes, this surge in vaping is concerning (Crotty Alexander et al., 2015; Perez and Crotty Alexander, 2020).

E-cigarettes were initially thought to have a role in smoking cessation, especially in the adult population. However, randomized control trials and observational studies have been largely inconclusive, and have predominantly found that smokers add vaping to their inhalant use, but continue using one or both inhalants in the long run (Bullen et al., 2013; Grana et al., 2014). Furthermore, on-going safety concerns surrounding the use of e-cigarettes call into question the utility of these devices as a treatment modality for tobacco cessation. For example, e-cigarette or vaping product use-associated lung injury (EVALI) is a novel disease caused by vaping, first recognized in 2019, and rapidly achieved epidemic proportions (Perrine et al., 2019). EVALI itself sickened thousands of Americans and led to the deaths of over 50 (Crotty Alexander et al., 2020). There are several other potential impacts of e-cigarettes on public health including renormalization of smoking behavior, tobacco dependence, and nicotine poisoning.

The toxicity of nicotine is well-established and known to have detrimental effects in the oral cavity and the lungs. However, the effects of non-nicotine components of e-cigarette aerosols on the oral cavity and airways are not yet clear. Studies have shown that exposure of lung epithelial cells to e-cigarette aerosols induces inflammation (measured by cytokines), causes oxidative damage, and impairs innate immune defenses (Lerner et al., 2015; Hwang et al., 2016; Crotty Alexander et al., 2018). Moreover, mouse models have shown that e-cigarette exposure increases the susceptibility to both viral and bacterial infections and decreases their clearance (Lerner et al., 2015; Corriden et al., 2020). However, there is a paucity of studies looking specifically at the oral microbiome, which plays a role in systemic and airway inflammation as well as in susceptibility to infection.

The oral cavity hosts a sophisticated and complex microbial community and is the primary gateway for microorganisms that colonize the lungs (Bowden, 2000; Dickson and Huffnagle, 2015; Mathieu et al., 2018). While inter‐ and intra-individual differences do exist, several studies have identified the common commensal bacterial phyla in the oral cavity, including *Firmicutes*, *Bacteroidetes*, *Proteobacteria*, *Actinobacteria*, *Spirochaetes*, and *Fusobacteria* (Zaura et al., 2009; Dewhirst et al., 2010). However, human habits can promote shifts in these microbes, leading to dysbiosis and disease (Hajishengallis et al., 2012). Although there are multiple studies on the effects of conventional tobacco products, e-liquids have multiple components (nicotine, THC, flavorings, propylene glycol, and glycerin) that are likely to have additional substantial impacts on the oral microbiome. For example, Pushalkar et al. (2020) studied the differences in the oral microbiome in e-cigarette users when compared to cigarette smokers and never smokers. They found that e-cigarette use modulated the microbiome and increased the abundance of specific bacteria (*Haemophilus*, *Fusobacteria*, and *Actinomyces*). This dysbiosis in microbial communes was associated with increased inflammation, as evidenced by increased cytokine release (Pushalkar et al., 2020). While informative, this study explored one oral sample type (i.e., saliva) at a static time period. Given that the oral microbiome can vary both spatially and temporally, it is imperative that additional analyses are conducted (Deo and Deshmukh, 2019).

Here, we recruited healthy, young non-smoking/non-vaping (NSNV) controls and healthy e-cigarette users to: (1) evaluate bacterial diversity and composition in the oral cavity (*via* saliva and buccal samples) of e-cigarette users relative to controls, (2) determine whether e-cigarette use induces persistent or acute changes by comparing samples collected before and after vaping reduction, and (3) utilize culture-based methodologies to compare nasal *Staphylococcus aureus* colonization rates in e-cigarette users and controls.

## Materials and Methods

### Recruitment of Subjects

University of California, San Diego (UCSD) IRB approved recruitment advertisements for 18–30-year-old e-cigarette users and NSNV controls were posted electronically on San Diego college websites. Paper versions were posted locally and electronic versions were posted on both Craigslist and Reddit. All responders to the advertisements underwent screening over the phone for inhalant use and exposure. No identifying information was obtained until tobacco, marijuana, and illicit drug screening was completed.

Controls were defined as young adults whom had never vaped e-cigarettes (more than once per month), smoked conventional cigarettes (none within one year and never more than once a month prior), use marijuana (MJ; more than once a month), or use any illicit drugs. These NSNV controls had one in-person clinic visit where samples were obtained. E-cigarette users were defined as subjects whom were active users of any e-device, including e-cigarettes, vape pens, box mods, pod-devices, or any other vaping device. Users had to consume ≥0.5–1 ml of e-liquid per day or 3.5–7 ml per week, for a period of at least 6 months to enroll in the study. To qualify as e-cigarette only users, subjects must not have smoked more than one cigarette per month for >6 months, no more than one use of MJ per month, with no illicit drug use. E-cigarette users had three separate in-person visits during which samples were acquired. Between visits two and three, e-cigarette users were asked to stop vaping for two weeks.

### E-cigarette Use

Demographic information of enrolled participants is shown in Table 1. Participants were asked to report their e-cigarette use patterns such as frequency, e-liquid preferences, and nicotine concentrations (Table 2). When reporting frequencies, responses were categorized based on the distribution. Device use length was categorized by ≤1, ≤2, and ≤3 years. Similarly, daily use (times/day) was categorized as ≤10, ≤20, ≤40, and >40 times/day. One participant who described vaping frequency as “entire day” was included in the maximum category. Although participants were provided nicotine replacement, in the form of gum, one of the e-cigarette users was able to fully stop vaping during the 2 weeks of intended cessation, but all participants reported decreased use over those 2 weeks (evaluated by questionnaire).

**Table 1:** Demographic characteristics of NSNV controls and e-cigarette users.

	Control (*n* = 12)	E-cigarette (*n* = 12)	*p*
Age (y)	21	21	0.6369
**Sex (*n*)**			
*M*	3	12	0.0003
*F*	9	0	
**Race/Ethnicity (*n*)**			
White (non-Hispanic)	1	3	0.6016
African American (non-Hispanic)	1	0	
Asian (non-Hispanic)	6	7	
Hispanic	2	1	
Unknown	2	1	

The Asian category included participants who self-reported as Chinese, Filipino, and Taiwanese. The Hispanic category included a participant who identified as Latino. An unpaired Mann Whitney test was performed for age, a two-sided Fischer’s exact test for sex, and a *χ*^2^ test for race/ethnicity. *p* ≤ 0.05 was considered significant.

**Table 2:** Self-reported descriptions of e-cigarette use.

	E-cigarette (*n* = 12)
Device use length (y)	1.5 (0.9–2.0)
≤1	4
≤2	7
≤3	1
Weekly use (days/week)	6.3 (5.7–7.0)
5	4
7	8
Daily use (times/day)	26.7 (12.0–41.3)
≤10	4
≤20	3
≤40	1
>40	4
**E-liquid type (PG/VG)**	
30/70	2
50/50	2
70/30	8
80/20	1
**Number of e-liquid flavors**	
1	7
2	1
3	1
Unknown	3
Nicotine concentrations (mg/ml)	21.3 (5.1–37.6)
≤3 mg/ml	4
4–6 mg/ml	4
≥50 mg/ml	4
Volume e-liquid per day (ml/day)	5.4 (0.6–12.2)
≤1	4
≤5	6
≥20	2

Means are presented with 95% CI.

Nicotine concentrations (mg/ml) were categorized as ≤3, 4–6, and ≥50 mg/ml. Categories for e-liquid volume (ml/day) were ≤1, ≤5, and ≥20 ml/day. Common e-liquid propylene glycol/vegetable glycerin (PG/VG) types were 30/70, 50/50, 70/30, and 80/20. One participant alternated between a PG/VG of 70/30 and 80/20, and was included in both categories.

### Sampling Procedures

For oral microbiome studies, NSNV controls and e-cigarette users (*n* = 12 each) were asked to not eat, drink, brush teeth, or chew gum prior to study visits, except for water. After obtaining informed consent, subjects were asked to rinse their mouths with water. After 10 min, subjects were asked to drool into a sterile sample container for 5 min and saliva volume was documented. Samples were centrifuged at 5000 *g* for 5 min prior to aliquoting and snap-freezing the supernatant. Buccal mucosa was then scraped gently with Omni Swab (Whatman) 6x, and the scraper head was ejected into 1 ml RNAlater (ThermoFisher) in a 2 ml cryovial. Samples were snap frozen and stored at −80°C for gene analysis.

For assessment of nasal colonization with *S. aureus*, 14 NSNV controls and 21 e-cigarette users had a nasal swab (BD) inserted into each nare and rotated 360° x2. The swab was then placed back into the swab container to assess for *S. aureus* colonization. The swab was used to streak Tryptic Soy Agar Plates with 5% Sheep’s Blood (Teknova), which were incubated at 37°C for 48 h. Colonies of *S. aureus* were frozen in glycerol.

### 16S rRNA Gene Amplicon Processing

For the buccal samples, swab tips were removed from cryovials under sterile conditions and subjected to total DNA extraction *via* the Qiagen DNeasy Powersoil kit (Qiagen; CA, United States). The same procedure was repeated for 200 μl aliquots of saliva. From the extracted DNA, the V3-V4 hypervariable region of the 16S rRNA gene was PCR amplified using Kapa Hifi Hotstart Readymix (Kapa Biosystems; Boston, MA, United States) with 16S Amplicon PCR Reverse Primer = 5' GTCTCGTGGGCTCGGAGATGTGTATAAGAGACAGGACTACHVGGGTATCTAATC 16S Amplicon PCR Forward Primer = 5' TCGTCGGCAGCGTCAGATGTGTATAAGAGACAGCCTACGGGNGGCWGCAG (Klindworth et al., 2013) using the following cycling parameters: 95°C for 3 min, followed by 35 cycles of 95°C for 30 s, 55°C for 30 s, 72°C for 30 s, and a final elongation step of 72°C for 5 min. An Ampure XP bead (Beckman-Coulter; Fullerton, CA, United States) cleanup step was then utilized to purify the resulting amplicons, which were visualized *via* the High Sensitivity DNA Kit on a Bioanalyzer (Agilent Technologies; Palo Alto, CA, United States) and quantified *via* the dsDNA High Sensitivity Kit on a Qubit Fluorometer (Thermo Fisher; United States). Samples were pooled into equal molar proportions and sequenced on the Illumina MiSeq platform (Illumina; San Diego, CA, United States). Negative extraction controls (sterile PBS) were included to ensure that no exogenous DNA contamination occurred during sample processing.

### Analysis of the 16S rRNA Gene Sequences

The resulting sequence reads were quality filtered and dereplicated using the DADA2 plugin in Quantitative Insights Into Microbial Ecology 2 (QIIME2; version 2019.7; Callahan et al., 2016; Bolyen et al., 2018). Alpha [observed operational taxonomic units (OTUs) and Faith’s phylogenetic diversity (PD)] and Beta (Bray Curtis Dissimilarity and Jaccard index) diversity metrics were produced by QIIME2 core-metrics-phylogenetic pipeline (sampling-depth parameter 19,000). Taxonomic classifications were generated using the qiime feature-classifier classify-sklearn feature, with a Naïve Bayes classifier trained on SILVA database and visualized at the genus level (Quast et al., 2012). Data were visualized using the qiime2R^1^ and ggplot2 packages in R-Studio (version 1.0.153; Faith, 1992; Wickham, 2009; Kolde and Kolde, 2018).

### Statistics

For demographics, we determined significance by using GraphPad Prism (version 8.4.1) to conduct an unpaired two-tailed Mann-Whitney test for age, a two-sided Fisher’s test for sex, and a *χ*^2^ test for race/ethnicity. Value of *p* less than 0.05 were considered statistically significant. Beta-diversity significance was determined using ANOSIM tests with 999 permutations. Linear discriminant analysis (LDA) effect size (LEfSe, Galaxy Version 1.0; Segata et al., 2011) was used to determine bacterial genera most likely to explain differences between the two cohorts and e-cigarette users before and after reduction in vaping. The parameters for these analyses were set with default value of *p*, *α* = 0.05, and an LDA score of 2.0. Significant differences between alpha diversity metrics and bacterial genera relative abundance were determined by either paired or unpaired Wilcoxon tests. FDR correction was used to correct for multiple hypothesis testing. All statistical analyses were conducted in R Studio (version 1.0.153).

## Results

### Demographics and E-cigarette Use Data

Participants were asked to report their demographic information including age, sex, race/ethnicity (Table 1). There was a significant difference in sex, with 100% of e-cigarette users being male, and 75% of controls being female (*p* < 0.001; Table 1). There were no other significant differences between the two cohorts.

E-cigarette users had an average device use length of 1.5 years, weekly use of 6.3 days/week, and a daily use of 26.7 times/day, with a maximum reported frequency of 60 times/day (Table 2). The most common PG/VG type was 70/30 (*n* = 8), and most participants used only one flavor, often mint or fruit. Nicotine concentrations ranged from 1.5 to 59 mg/ml with an average of 21.3 mg/ml. E-liquid volume use ranged from 0.7 to 30 ml/day, with an average of 5.4 ml/day. The most common reported e-cigarette brands were SMOK, Juul, and Suorin, while the most common e-liquid brand was Naked 100.

### Sequencing Output

After DNA extraction, 16S rRNA amplification/sequencing, and quality filtering 71 oral samples were included in the analyses for a total of 3,097,305 sequence reads with an average number of 43,624 sequences per sample ±19,480 standard deviation (S.D.) To account for unequal sequencing depth, data were normalized to an even sampling depth of 19,000 sequences per sample. Both saliva and buccal samples were collected from the same 12 NSNV controls. Similarly, 12 e-cigarette users were included in the study; however, two e-cigarette users did not achieve the minimum 3 ml volume for their saliva samples. Additionally, for both sample types, there were nine paired samples collected before and after decreased e-cigarette use for 2 weeks. Only one unique sample per participant was utilized to compare cohorts and only paired samples were used to elucidate changes associated with reduced e-cigarette product use.

### Alpha and Beta Diversity Among E-cigarette Users and NSNV Controls

**Figure 1:**
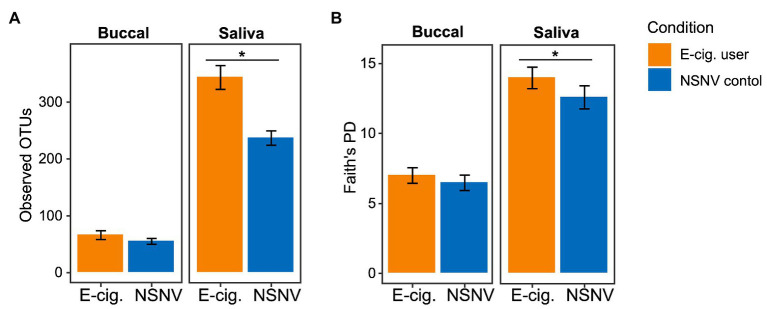
Alpha diversity bar plot showing **(A)** observed operational taxonomic units (OTUs; ±SE) and **(B)** Faith’s phylogenetic diversity (PD; ±SE) from buccal and saliva samples collected from electronic (e)-cigarette users and non-smokers/non-vapers (NSNV) controls. The alpha diversity indices are shown on the *y*-axis and the e-cigarette status (E-cigarette users, orange; NSNV controls, blue) is on the *x*-axis. Significant difference (*p* < 0.05) determined by an unpaired two-sample Wilcoxon test.

To determine any potential differences in alpha diversity among the two cohorts, we examined several metrics including those that assess richness (Observed OTUs) and phylogenetic biodiversity (Faith’s PD). Of these, we observed a significantly (Wilcoxon test; *p* < 0.05) higher number of Observed OTUs and Faith’s PD in saliva from e-cigarette users compared to NSNV controls (Figure 1).

**Figure 2:**
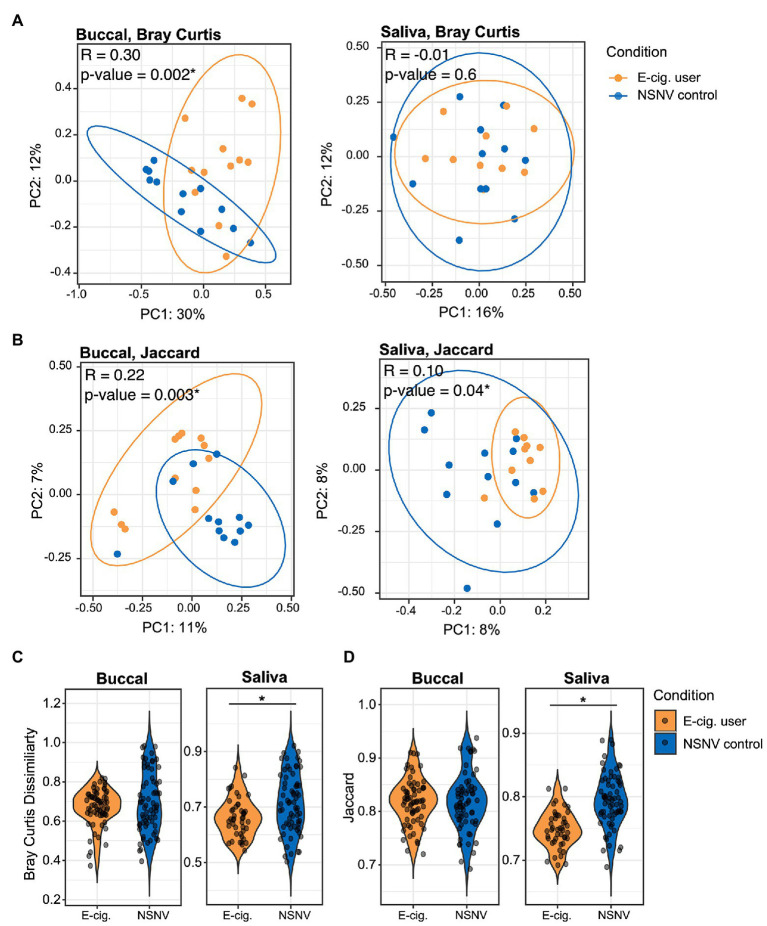
Beta diversity of e-cigarette users and NSNV controls. **(A,B)** Principal coordinates plots of beta-diversity based on **(A)** Bray Curtis and **(B)** Jaccard indices from buccal and saliva samples collected from e-cigarette users and NSNV controls. Color denotes e-cigarette status (E-cigarette users, orange; NSNV controls, blue). Ellipses are drawn at 95% CI for e-cigarette status. Significance determined by ANOSIM with 999 permutations for e-cigarette status and denoted in the upper corner of each panel, ^*^p < 0.05. **(C,D)** Violin plots showing the distribution of **(C)** Bray Curtis and **(D)** Jaccard indices within each cohort (e-cigarette users and NSNV controls) from buccal and saliva samples. Significant difference (*p* < 0.05) determined by an unpaired two-sample Wilcoxon test.

We next quantified beta diversity using two metrics, Bray Curtis Dissimilarity and the Jaccard index. Both use presence/absence data to estimate the difference between communities either with (Bray Curtis) or without abundance (Jaccard) information. A clear and significant clustering was apparent between the two cohorts in the buccal samples for both metrics (Figures 2A,B, ANOSIM; *p* < 0.05), indicating the delineation of the two cohorts. Significant clustering was only observed in the saliva samples for the Jaccard index. Additionally, for the saliva samples, the Bray Curtis and Jaccard indices within e-cigarette users were significantly smaller than within NSNV controls (Figures 2C,D, Wilcoxon test; *p* < 0.05), that is, the saliva of e-cigarette users are more similar to each other than the NSNV controls are to each other.

### Alterations in Oral Bacterial Taxonomic Composition in E-cigarette Users and NSNV Controls

**Figure 3:**
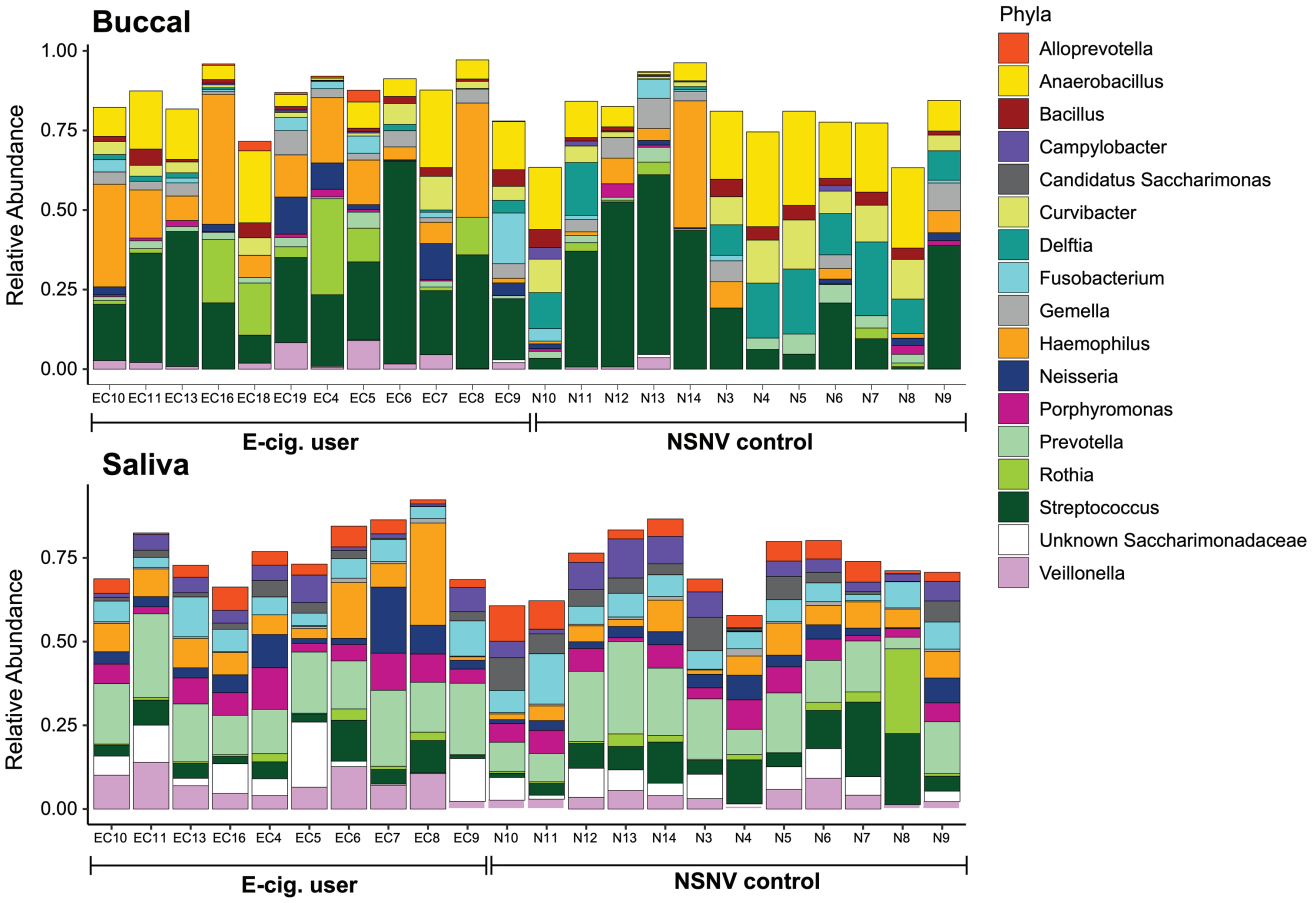
Stacked bar chart of the relative abundance of the bacterial community composition from buccal and saliva samples for each subject. The relative abundance of each of the dominant bacterial families is shown on the *y*-axis and the subject ID is on the *x*-axis grouped by e-cigarette status.

**Figure 4:**
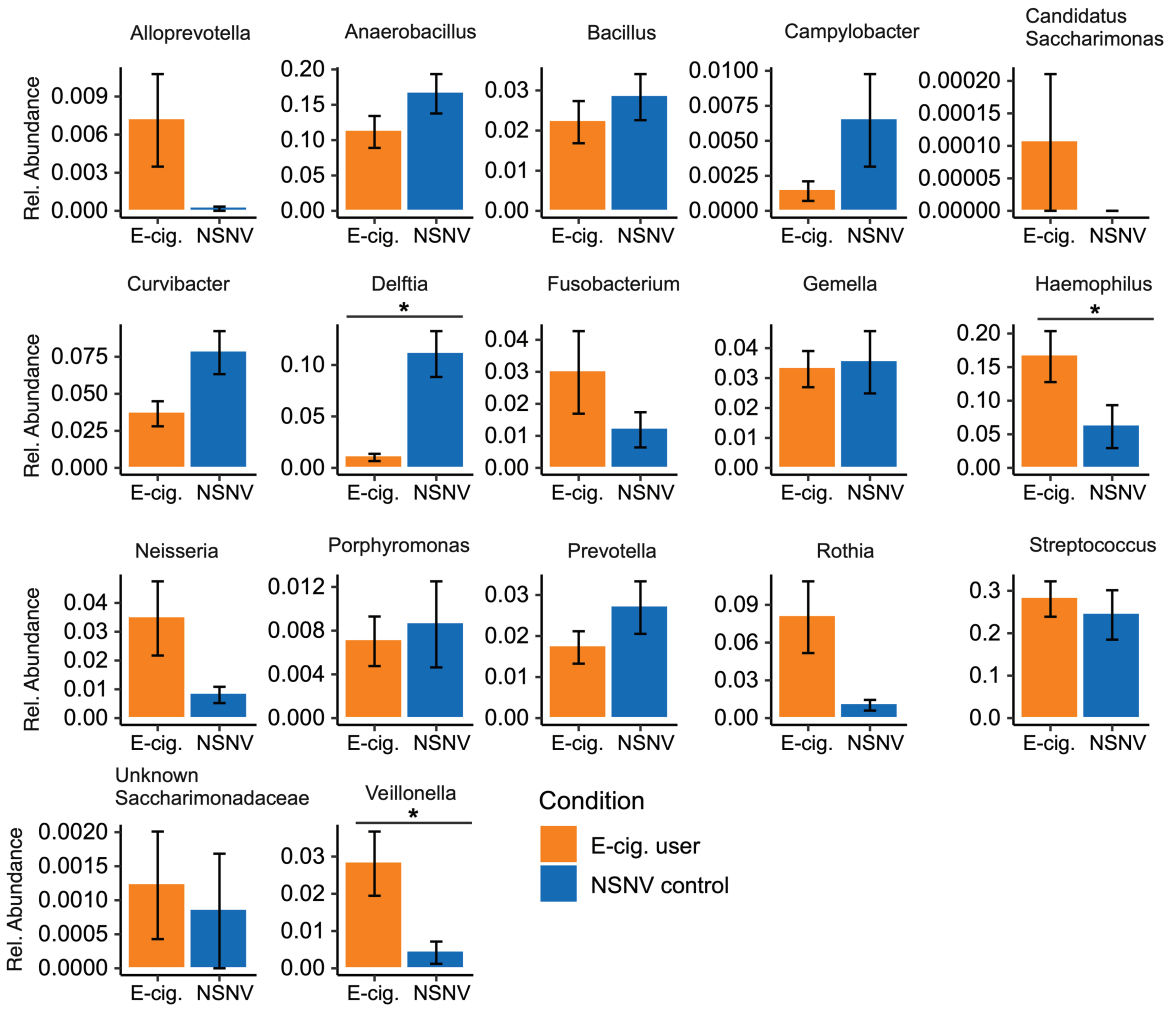
Bar charts of the relative abundance (±SE) of the dominant bacterial community taxa present in e-cigarette users and NSNV controls for buccal samples. For each of the dominant bacterial taxa, the relative abundance is listed on the *y*-axis and the e-cigarette status (E-cigarette users, orange; NSNV controls, blue) is on the *x*-axis. Significant difference (*p* < 0.05) determined by an unpaired two-sample Wilcoxon test with FDR correction.

We next explored the dominant bacterial genera (genera present at ≥5% in at least five samples) in both the buccal and saliva samples (Figure 3). In the buccal samples, *Streptococcus* was on average the most abundant bacteria genera in both e-cigarette users (28 ± 14% S.D.) and NSNV controls (24 ± 20% S.D.), whereas *Prevotella* was on average the most abundant in the saliva samples for both e-cigarette users (18 ± 5% S.D.) and NSNV controls (15 ± 7% S.D.). However, there was a large degree of individual variability even within each cohort (Figure 3). Despite this, significant differences were observed when examining the cohorts in aggregate (Figure 4). For the buccal samples, the relative abundance of *Veillonella* and *Haemophilus* were significantly higher in the e-cigarette users compared to the NSNV controls (Figure 4, Wilcoxon test; *p* < 0.05). However, in the saliva samples, there were no genera that were significantly different between the two cohorts (Supplementary Figure S1).

**Figure 5:**
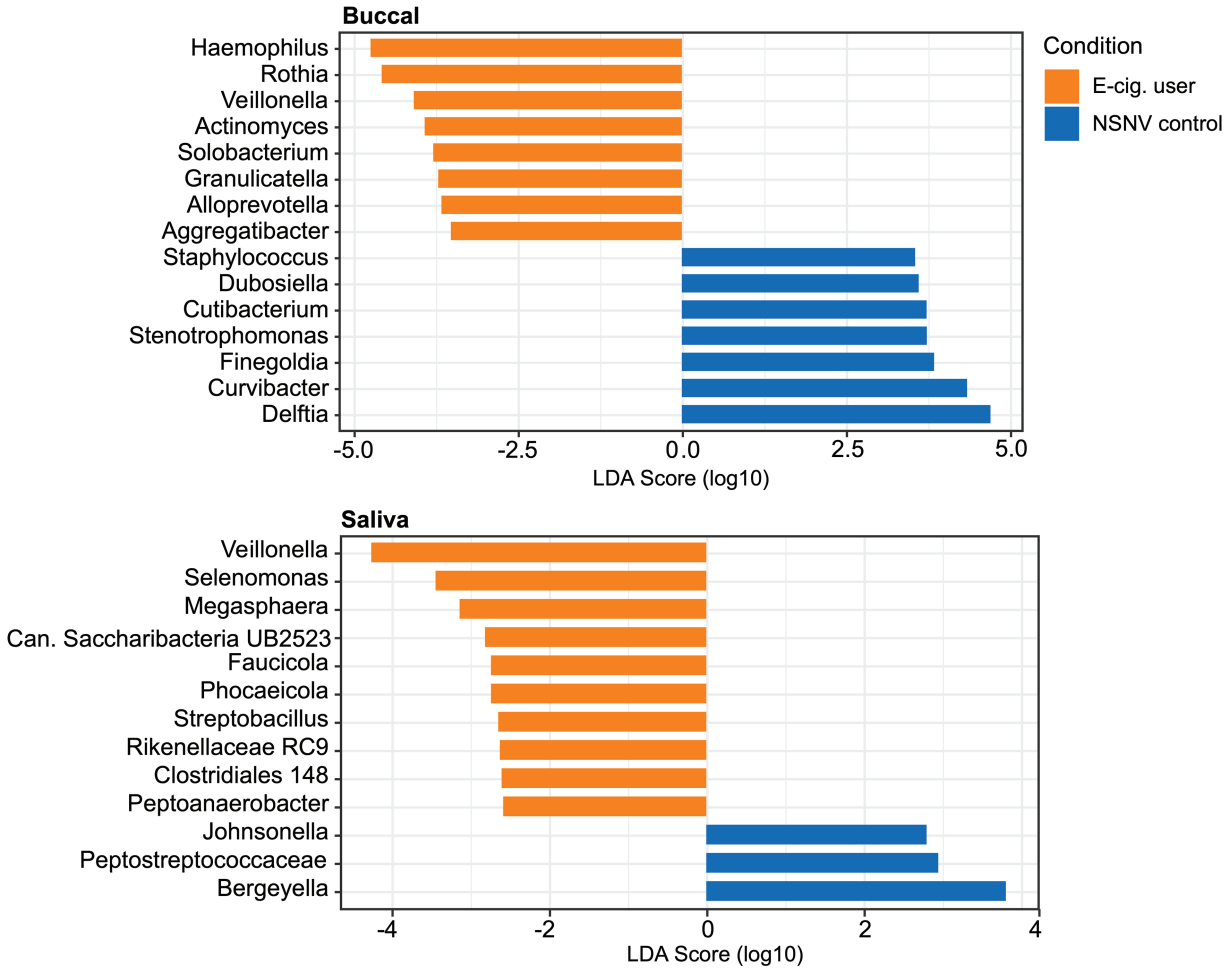
Linear discriminant analysis (LDA) scores (*α* = 0.05, LDA score > 2.0) computed for differentially abundant bacterial taxa between e-cigarette users and NSNV controls from buccal and saliva samples. The log10 transformed LDA scores are showed on the *x*-axis and the bacterial taxa are listed on the *y*-axis. Orange bars indicate the taxa found in greater relative abundance in e-cigarette users. Blue bars indicate taxa found in greater relative abundance in NSNV controls.

To further assess the degree of variation between the two cohorts, we performed a LEfSe analysis to identify important taxonomic differences between e-cigarette users and NSNV controls for both the buccal and saliva samples (Figure 5). Based on this analysis, we identified several notable differences between the two cohorts in both sample types. Specifically, in the buccal samples, we observed that the relative abundance of *Haemophilus*, *Rothia*, *Veillonella*, *Actinomyces*, *Solobacterium*, *Granulicatella*, *Alloprevotella*, and *Aggregatibacter* was enriched in the e-cigarette users compared to the NSNV controls. Similarly, *Veillonella* was also enriched in the saliva of e-cigarette users along with *Selenomonas*, *Megasphaera*, *Candidatus Saccharibacteria UB2523*, *Faucicola*, *Phocaeicola*, *Streptobacillus*, *Rikenellaceae RC9 group*, *Clostridiales group 148*, and *Peptoanaerobacter*.

### Variation in Community Composition and Diversity After Reduction in E-cigarette Use

**Figure 6:**
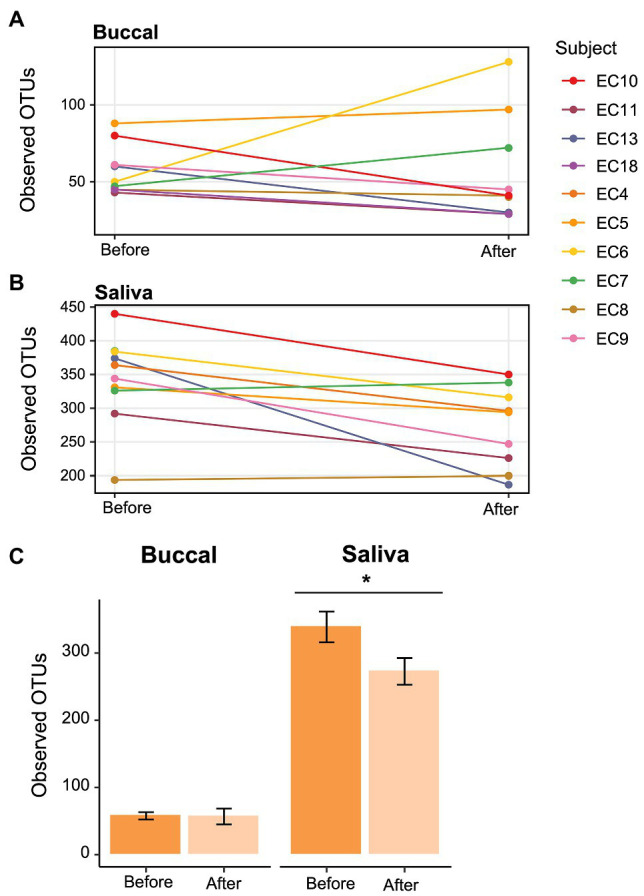
Change in alpha diversity in e-cigarette users at each visit. **(A,B)** Line graph showing the number of Observed OTUs from **(A)** buccal and **(B)** saliva samples collected from e-cigarette users before and after reduction in vaping. The Observed OTUs are shown on the *y*-axis and the time before and after reduction in vaping on the *x*-axis. **(C)** Bar plots showing the aggregate data (±SE) before and after reduction in product use for buccal and saliva samples. Significance (*p* < 0.05) determined by a paired two-sample Wilcoxon test.

To determine whether the effects of e-cigarette use on the oral microbiome were acute or chronic, participants in the e-cigarette cohort were asked to decrease use of all e-cigarette and vaping devices. We then compared the visits before and after decreased e-cigarette use to determine acute e-cigarette effects. When looking at the number of Observed OTUs between the visits, we found that after decreasing e-cigarette use, subjects had a significantly (Paired Wilcoxon test; *p* < 0.05) lower number of Observed OTUs compared to their initial visit (Figure 6). When comparing the samples collected after decreased e-cigarette use with NSNV controls for both buccal and saliva samples, there were no longer any significant differences in the number of Observed OTUs or Faith’s PD (Wilcoxon test; *p* < 0.05). This suggests that reduction of e-cigarette use may reverse the increase in diversity observed in e-cigarette users (Figure 1A).

As for the taxonomic composition, we observed no significant changes in the dominant bacterial genera before and after decreased e-cigarette use (Supplementary Figures S2, S3). However, when performing a LEfSe analysis between the visits in the buccal samples, two genera were determined to be differentially abundant; the relative abundance of *Halomonas* (LDA 3.7) was higher prior to decreased e-cigarette use and *Stenotrophomonas* (LDA: 3.8) was higher after. For the saliva samples, *Delftia* (LDA: 2.7), *Lachnoanaerobaculum* (LDA: 2.7), and *Johnsonella* (LDA: 2.7) were at a higher relative abundance after decreasing e-cigarette use. This lack of shift in the taxonomy may be due to the change in inter-individual diversity. For both sample types, the Jaccard distances within e-cigarette users were significantly smaller during regular e-cigarette vaping, suggesting that the inter-individual diversity among the e-cigarette users increased when the subjects decreased e-cigarette use to varying degrees (Supplementary Figure S4; Wilcoxon test; *p* < 0.05).

### Nasopharyngeal Colonization With *Staphylococcus aureus*

One of 14 NSNV control subjects was colonized with *S. aureus* by nasal swab, while four of 21 e-cigarette users were colonized (7.1 vs. 19%). The demographics for these larger cohorts were not significantly different than the focused cohorts in the oral microbiome studies (Supplementary Tables S1, S2).

## Discussion

The oral cavity provides optimal conditions for the proliferation and survival of a complex microbial ecosystem (Bowden, 2000). However, human habits, like vaping, can change the chemistry and composition of the oral cavity (Pavlova et al., 1997; Pavlova and Tao, 2000; Morris et al., 2013; Thomas et al., 2014; Mason et al., 2015). *In vitro* and *in vivo* studies on the effects e-cigarette aerosol exposure have shown increased markers of oxidative stress, inflammation, DNA strand breakage, and damage to the oral tissues (Yu et al., 2016; Chaffee, 2019). In our study, we found that e-cigarette users had a significantly different oral microbiome composition compared to NSNV controls. This suggests that e-cigarette use may result in dysbiosis of the oral commensal microbial communities, a state often associated with systematic disease.

In the saliva of e-cigarette users, we observed a significantly higher alpha diversity compared to NSNV controls (Figure 1). These results are consistent with a previous study utilizing 16S rRNA sequencing to compare e-cigarette users with controls, as well as studies evaluating the oral microbial environment of traditional tobacco users (Kumar et al., 2011; Pushalkar et al., 2020). Moreover, this expansion of the oral biodiversity is also associated with periodontitis, an inflammatory disease linked to the microbiome. This disease is common in smokers and, more recently, in e-cigarette users (Liu et al., 2012; Shi et al., 2018; Genco et al., 2019). A longitudinal study found that participants who used vaping products regularly had increased odds of being diagnosed with gum disease and bone loss around the teeth (Atuegwu et al., 2019). The deepening and increasing periodontal pockets associated with periodontitis may be one of the contributors to the elevated biodiversity observed here and in previous studies, as it can provide a novel niche for specific bacteria able to withstand the limited-oxygen environment (Linden and Mullally, 1994; Griffen et al., 2012).

Interestingly, we only observed a greater degree of biodiversity among e-cigarette users in the saliva and not in the buccal samples. This may be due to the heterogeneous nature of saliva. Saliva contains a wide range of bacterial species, shed from the distinctive microenvironments of teeth, gingival crevices, tongue, and buccal/palatal mucosa (Costalonga and Herzberg, 2014). As a result, this aggregate of bacterial communities may better lend to the resolution of systematic differences in the oral cavity. Conversely, the buccal mucosa is far less diverse and far more distinctive, likely due to the specificity between the bacterial surface adhesins and the buccal surface receptors (Nobbs et al., 2011). Because of this specificity, however, we were able to better resolve potential differences in the taxonomy of the bacterial community between the two cohorts in the buccal samples. For instance, clear and significant clustering was apparent between the e-cigarette users and NSNV controls in the buccal samples (Figures 2A,B).

Additionally, using two methodologies, we found that *Veillonella* was significantly higher in relative abundance in the buccal samples of e-cigarette users (Figures 4, 5). This is in agreement with a previous study, which found that *Veillonella atypica* and *Veillonella rogosae* were highly enriched in e-cigarette and combustible cigarette users compared to healthy controls (Pushalkar et al., 2020). *Veillonella* are common residents of the human oral cavity and gastrointestinal tract (Rogosa, 1964). While they are generally considered commensal, some species have been associated with infections of the mouth, soft tissues, sinuses, lungs, heart, bones, and central nervous system (Brook, 1996; Bhatti and Frank, 2000). While not always consistent, *Veillonella* spp. were reported to be enriched in the subgingival plaque (Moon et al., 2015), right and left oropharynx (Charlson et al., 2010), small intestinal mucosa (Shanahan et al., 2018), sputum (Lim et al., 2016), and saliva (Al-Zyoud et al., 2020) of cigarette smokers compared to controls. *Veillonella* are also reported to be dominant species in the subgingival biofilm samples of patients with chronic periodontitis and have been detected (with species-specific primers) at a higher rate in subjects with poor oral hygiene compared to those with good or moderate oral hygiene (Gross et al., 2012; Mashima et al., 2016). Furthermore, due to their ability to convert nitrate to nitrite, some *Veillonella* species have been suggested to play a role in the formation of tobacco specific nitrosamines (TSNAs; Kato et al., 2016; Pushalkar et al., 2020), carcinogens derived from the nitrosation of tobacco alkaloids (Preston-Martin, 1991; Atawodi and Richter, 1996; Gupta et al., 1996).

In addition to *Veillonella*, *Haemophilus* was at a significantly higher relative abundance in the buccal samples of e-cigarette users compared to controls (Figures 4, 5). Like *Veillonella*, *Haemophilus* spp. are common to the oral cavity and upper respiratory tract. However, *Haemophilus influenza*, specifically, is widely known for its both direct and indirect (bacteria-mediated inflammation) contribution to smoking-associated lung disease (King, 2012; Faner et al., 2017). In fact, *H. influenza* is the most common bacteria found in the lower airways of patients with chronic obstructive pulmonary disease (COPD; Finney et al., 2014; Sriram et al., 2018). Exposure of *H. influenza* isolates to e-cigarette aerosols *in vitro* has also been shown to increase the degree of biofilm formation, a process that could aid in the establishment of persistent infection (Gilpin et al., 2019). Moreover, *H. influenza* isolates exposed to e-cigarette aerosols were determined to provoke a significantly greater inflammatory response in human airway epithelial A549 cells compared to non-exposed bacterial cells (Gilpin et al., 2019).

To further our analyses of microbial profiles of e-cigarette users, we sought to determine the effect of short-term reduction of e-cigarette and vaping product use. It has been previously demonstrated that traditional tobacco smoking cessation alters the microbiome of the oral cavity, with restoration of the oral microbiome occurring relatively rapidly after smoking cessation (1–2 years; Wu et al., 2016). When comparing the visits before and after decreased e-cigarette use, we found that there was a significant decrease in alpha diversity (Figure 6). In fact, both alpha diversity metrics (Observed OTUs and Faith’s PD) were no longer significantly different between e-cigarette users after decreased use and the NSNV controls. This suggests that the increased diversity, putatively caused by e-cigarette use, may be mitigated following even short-term cessation.

Interestingly, there were no significant changes in the major bacterial genera in either the saliva or buccal samples following diminished e-cigarette use (Supplementary Figures S2, S3). We hypothesize that this may be due to movement away from the diseased state to one that is more individual specific. We found that inter-individual diversity in the saliva of NSNV controls was greater than that of e-cigarette users, and after decreased e-cigarette use inter-individual diversity among the e-cigarette users increased significantly in the buccal and saliva samples (Figure 2; Supplementary Figure S4). This is in agreement with a previous study that reported a greater variability in the microbiome of participants in good oral health compared to those with periodontitis (Liu et al., 2012). The authors ascribed this to the disease state occupying a narrow region within the space of possible microbiome configurations; a diverse population adapted to the diseased environment (Liu et al., 2012). Here, the increased alpha diversity and lower beta diversity of e-cigarette users may be caused by a disrupted host homeostasis and a “cloud” of opportunistic scavengers able to make use of the by-products of vaping.

Shifts in the nasopharyngeal microbiome are also known to occur in response to inhalant exposure, with increased presence of known human pathogens. Conventional tobacco smokers have higher rates of *S. aureus* nasopharyngeal colonization, which is believed to be one of the driving factors behind the increased rates of *S. aureus* infections in smokers. Increased colonization has been tied to direct effects of tobacco smoke both on human cells and directly on the virulence of *S. aureus* (Hwang et al., 2016). E-cigarette aerosols have also been found to drive virulence in *S. aureus* and diminish host defenses in the airways (Hwang et al., 2016; Corriden et al., 2020). Thus, we evaluated for *S. aureus* colonization as another airway microbial assessment. While the results did not reach statistical significance, we did observe that e-cigarette users had a higher colonization rate of nasal *S. aureus* relative to controls (19 vs. 7.1%). Future studies of larger cohorts, powered to detect changes in colonization rates of airway pathogens, are advised, as higher rates of colonization are associated with higher rates of invasive disease.

E-cigarettes have been promoted by some to be a safer alternative to conventional tobacco cigarettes; however, there remains a tremendous lack of data as to the chronic health effects that these diverse nicotine drug delivery devices will cause. We identified multiple changes that are in-line with those seen in cigarette smokers and patients with gum disease. These changes included variations in the microbial community composition (e.g., *Veillonella* and *Haemophilus* abundance) and a greater alpha diversity compared to NSNV controls. However, this study had limitations. The most obfuscating factor regarding any study of the human microbiome is the degree of individual variation. There are factors that may have significant contributions to the oral microbiome that were not included in this study, such as diet, alcohol consumption, oral hygiene, and even host genetics (e.g., physical properties of the oral landscape, immune response; Kilian et al., 2016; Murtaza et al., 2019; Ulloa et al., 2019). Additionally, we were unable to match the cohorts based on sex; with the e-cigarette cohort having significantly more males (Table 1). While there are a limited number of studies that have addressed the potential interaction between sex and the oral microbiome in adults, there is some evidence to suggest that sex influences the composition and diversity of the microbiome, particularly in the gut (Mueller et al., 2006; Li et al., 2008; Sinha et al., 2019). However, these results are not always consistent, with multiple studies showing no differences in the microbiome based on sex (Schenkein et al., 1993; Kim et al., 2020). Considering that some of the significant results observed here were diminished when the participants were asked to reduce vaping, it is likely that sex was not the prominent driver of the differences between e-cigarette users and NSNV controls. Future studies should seek to control for these and other confounders.

As adolescents and young adults pick up the habit of vaping e-cigarettes, become addicted and expose their upper airways to e-cigarette aerosols chronically, it becomes more imperative to understand the potential impact on the oral microbiome, and ultimately human health. This work, though limited by a small number of subjects, identifies shifts in the microbes present in the nose and mouth that indicate pathologic changes induced by e-cigarette use.

## Data Availability Statement

The datasets presented in this study can be found in online repositories. The names of the repository/repositories and accession number(s) can be found at: https://www.ncbi.nlm.nih.gov/, PRJNA622970.

## Ethics Statement

The studies involving human participants were reviewed and approved by University of California, San Diego (UCSD) IRB. The patients/participants provided their written informed consent to participate in this study.

## Author Contributions

LC and DP: conception and design of the experiments. JC, CB, JS, AM, AF, SB, DC, DP, and LC: acquisition, analysis, and interpretation of data. JC, CB, DC, SB, DP, and LC: manuscript composition. All authors contributed to the article and approved the submitted version.

### Conflict of Interest

The authors declare that the research was conducted in the absence of any commercial or financial relationships that could be construed as a potential conflict of interest.

## Footnotes

^1^https://github.com/jbisanz/qiime2R
