# Supplementary material for: Compositional Differences in the Oral Microbiome of E-cigarette Users
Source: Front Microbiol. 2021 May 31;12:599664. doi: 10.3389/fmicb.2021.599664 (PMC8200533; doi:10.3389/fmicb.2021.599664)
Supplement: Supplementary file 1 [file Data_Sheet_1.DOCX]

**Supplemental Figures and Tables**

**Figure S1**: Bar charts of the relative abundance (±standard error) of the dominant bacterial community taxa present in e-cigarette users and NSNV controls for saliva samples. For each of the dominant bacterial taxa the relative abundance is listed on the y-axis and the e-cigarette status (E-cigarette users, orange; NSNV controls, blue) is on the x-axis. Significant difference (*p* < 0.05) determined by an unpaired two-sample Wilcoxon test with FDR correction.

**Figure S2:** Bar charts of the relative abundance (±standard error) of the dominant bacterial community taxa present in e-cigarette users buccal samples before and after reduction in vaping. For each of the dominant bacterial taxa the relative abundance is listed on the y-axis and the e-cigarette visit is on the x-axis. Significant difference (p < 0.05) determined by a paired two-sample Wilcoxon test with FDR correction.

**Figure S3:** Bar charts of the relative abundance (±standard error) of the dominant bacterial community taxa present in e-cigarette users saliva samples before and after reduction in vaping. For each of the dominant bacterial taxa the relative abundance is listed on the y-axis and the e-cigarette visit is on the x-axis. Significant difference (p < 0.05) determined by a paired two-sample Wilcoxon test with FDR correction.

**Figure S4**: Violin plots showing the distribution of (A) Bray Curtis and (B) Jaccard indices within e-cigarette users before and after reduction in vaping from buccal and saliva samples. Significant difference (*p* < 0.05) determined by a paired two-sample Wilcoxon test with FDR correction.

**Table S1:** Demographic characteristics of all recruited subjects: NSNV (non-smoking/ non-vaping) controls and e-cigarette users. Participants who identified as Korean, Chinese, Filipino, and Taiwanese were included in the Asian category. Participants who identified as Asian Hispanic, Caucasian Hispanic, and Latino were included in the Hispanic category. An unpaired Mann Whitney test was performed for age, a two-sided Fischer's exact test for sex, and a χ2 test for race/ethnicity. P ≤ 0.05 was considered significant.

|  | **Control (n=14)** | **E-cig.**  **(n=21)** | **P-value** |
| --- | --- | --- | --- |
| **Age (y)** | 22 | 21 | 0.5287 |
| **Sex (n)** |  |  | < 0.0001 |
| M | 4 | 20 |  |
| F | 10 | 1 |  |
| **Race/Ethnicity (n)** |  |  | 0.3868 |
| White (non-Hispanic) | 1 | 6 |  |
| African American (non-Hispanic) | 1 | 0 |  |
| Asian (non-Hispanic) | 7 | 10 |  |
| Hispanic | 3 | 3 |  |
| Unknown | 2 | 1 |  |
| Mixed | 0 | 1 |  |

**Table S2:** Self-reported descriptions of e-cigarette use of all recruited subjects. Means are presented with 95% confidence intervals. One participant used a PG/VG (propylene glycol/vegetable glycerin) of 70/30 and 80/20.

|  | **E-cig. (n=21)** |
| --- | --- |
| **Device use length (y)** | 1.4 (0.9-1.9) |
| ≤ 1 | 11 |
| ≤ 2 | 7 |
| ≤ 3 | 2 |
| ≤ 4 | 1 |
| **Weekly use (days/wk)** | 6.1 (5.6-6.6) |
| < 5 | 2 |
| 5-6 | 7 |
| 7 | 12 |
| **Daily use (times/day)** | 22.3 (13.0-31.6) |
| ≤ 10 | 8 |
| ≤ 20 | 6 |
| ≤ 40 | 2 |
| > 40 | 5 |
| **E-liquid type (PG/VG)** |  |
| 30/70 | 6 |
| 50/50 | 4 |
| 70/30 | 11 |
| 80/20 | 1 |
| **Number of e-liquid flavors** |  |
| 1 | 14 |
| 2 | 2 |
| 3 | 2 |
| Unknown | 3 |
| **Nicotine concentrations (mg/ml)** | 24.2 (12.7-38.7) |
| ≤ 3mg/ml | 7 |
| 4 – 6 mg/ml | 5 |
| 7– 20 mg/ml | 1 |
| ≥ 50 mg/ml | 8 |
| **Volume e-liquid per day (ml/day)** | 4.4 (1.1- 7.7) |
| ≤ 1 | 10 |
| ≤ 5 | 9 |
| ≥ 20 | 2 |
